# Differentiation of Mouse Induced Pluripotent Stem Cells (iPSCs) into Nucleus Pulposus-Like Cells *In Vitro*

**DOI:** 10.1371/journal.pone.0075548

**Published:** 2013-09-25

**Authors:** Jun Chen, Esther J. Lee, Liufang Jing, Nicolas Christoforou, Kam W. Leong, Lori A. Setton

**Affiliations:** 1 Department of Orthopaedic Surgery, Duke University Medical Center, Durham, North Carolina, United States of America; 2 Department of Biomedical Engineering, Duke University, Durham, North Carolina, United States of America; Ohio State University, United States of America

## Abstract

A large percentage of the population may be expected to experience painful symptoms or disability associated with intervertebral disc (IVD) degeneration – a condition characterized by diminished integrity of tissue components. Great interest exists in the use of autologous or allogeneic cells delivered to the degenerated IVD to promote matrix regeneration. Induced pluripotent stem cells (iPSCs), derived from a patient’s own somatic cells, have demonstrated their capacity to differentiate into various cell types although their potential to differentiate into an IVD cell has not yet been demonstrated. The overall objective of this study was to assess the possibility of generating iPSC-derived nucleus pulposus (NP) cells in a mouse model, a cell population that is entirely derived from notochord. This study employed magnetic activated cell sorting (MACS) to isolate a CD24^+^ iPSC subpopulation. Notochordal cell-related gene expression was analyzed in this CD24^+^ cell fraction via real time RT-PCR. CD24^+^ iPSCs were then cultured in a laminin-rich culture system for up to 28 days, and the mouse NP phenotype was assessed by immunostaining. This study also focused on producing a more conducive environment for NP differentiation of mouse iPSCs with addition of low oxygen tension and notochordal cell conditioned medium (NCCM) to the culture platform. iPSCs were evaluated for an ability to adopt an NP-like phenotype through a combination of immunostaining and biochemical assays. Results demonstrated that a CD24^+^ fraction of mouse iPSCs could be retrieved and differentiated into a population that could synthesize matrix components similar to that in native NP. Likewise, the addition of a hypoxic environment and NCCM induced a similar phenotypic result. In conclusion, this study suggests that mouse iPSCs have the potential to differentiate into NP-like cells and suggests the possibility that they may be used as a novel cell source for cellular therapy in the IVD.

## Introduction

The healthy intervertebral disc (IVD) relies upon the well hydrated and proteoglycan-rich nucleus pulposus (NP) tissue to support and distribute the loads of spinal mobility and joint loading [1,2]. The immature nucleus pulposus contains more than 85% water, and a high density of randomly organized type II collagen fibers with lesser amounts of collagen types III, V, VI, and IX, elastin, and laminins type 111, 511 and 332 [3-8]. This compositionally unique extracellular matrix (ECM) is generated and maintained by a unique population of NP cells which express phenotypic markers that suggest their notochordal origin, including specific cytokeratins, vimentin, transcription factor (Brachyury, T) and cell surface marker (CD24) [9-14]. While this NP cell phenotype is associated with development and growth, there may be a shift towards a more sparse population of chondrocyte-like cells in the NP with aging [15]. IVD function may become compromised with aging-associated degeneration or in pathologies such as IVD herniation, processes that are associated with loss of disc height, decreased hydration, and a dramatic loss of cellularity believed to be key to the progressive nature of IVD pathology [16]. IVD disorders may contribute to pain and disability is a large number of patients, afflicting over 80% of adults and responsible for a socioeconomic toll of $100 billion annually in the United States alone [16-18]. These staggering consequences prompt a better understanding of the mechanisms governing IVD pathology, and more importantly, the invention of strategies that would stimulate its repair.

Cell-based tissue regeneration has emerged as an area of tremendous interest, with studies reporting matrix regenerative potential for many cell sources, including autologous chondrocytes, primary IVD cells and stem cells [19-21]. The question of cell source is of particular importance for cell-based IVD regeneration, given that the availability of autologous disc cells is extremely low in the adult, and that the mature adult phenotype may differ substantially from that of the immature IVD cell. In early work, autologous or allogeneic NP cells were isolated, expanded and re-implanted at high cell numbers in animal IVDs, demonstrating some beneficial effects in inhibiting the degenerative changes of nucleotomy [22-25]. Autologous disc cell transplantation has also been evaluated in clinical trials for follow-up treatment to discectomy [26], leading to the emergence of clinical products and platforms that support autologous cell supplementation to the IVD. Given the very limited availability of native and healthy IVD cells that can be harvested for therapy, however, there has been interest in using stem cell sources with a particular focus on bone marrow-derived mesenchymal stem cells (MSCs) [27,28] as well as adult stem cells [29,30]. The *in vitro* differentiation of MSCs into NP-like or chondrocyte-like cells has been demonstrated under hypoxic and high osmotic pressure conditions, along with transforming growth factor (TGF)-β and notochordal cell conditioned medium stimulation [28,31,32]. In those studies, limited knowledge of unique NP phenotypic markers has impaired a clear demonstration of the MSC differentiation potential into an NP-like cell lineage [33,34]. Preclinical studies have followed injection of autologous MSCs embedded in atelocollagen gel as well as direct injection of MSCs into rabbit or rat models of IVD degeneration, and detected an ability of these cells to differentiate or regenerate a hydrated and proteoglycan-rich matrix [35,36]. These findings have supported the expanded use of autologous MSCs in clinical trials for IVD disorders, despite adverse effects associated with donor site harvest and *ex vivo* cell expansion. Consequently, the need to identify additional cell sources supportive of regeneration of NP-like tissue remains of great interest.

In 2006, Takahashi and Yamanaka demonstrated that mouse embryonic fibroblasts could be reprogrammed into pluripotent stem cells via retroviral transfection of four transcription factors: *Oct3/4, Sox2, c-Myc* and *Klf4* [37]. This was subsequently proven feasible in fibroblasts from humans [38], rhesus monkeys [39], pigs [40], and rats [41]. Induced pluripotent stem cells (iPSCs) resemble embryonic stem cells (ESCs) functionally, differentiating into cells from all three germ layers – ectoderm, endoderm, and mesoderm. iPSCs alleviate ethical concerns tied to usage of ESCs, in addition to representing a possible autologous source for individualized treatments. It is well recognized now that iPSCs derived from a patient’s own somatic cells represent an attractive cell source, having the potential to differentiate into various cell types including neurons [42,43], cardiomyocytes [44,45], and cells of a hematopoietic lineage [46]. Of relevance to the proposed work are studies demonstrating the ability to promote differentiation of mouse or human iPSCs into chondrocyte-like cells, facilitated by supplemental growth factors that include bone morphogenetic proteins (BMPs) or TGFs [47-49]. These studies have demonstrated an ability for differentiated iPSCs to synthesize and express type II collagen and aggrecan – key components of both cartilage and NP ECM. These findings highlight the potential of using iPSCs as a source for generating differentiated NP-like cells, but also point out a need for identifying those factors that can promote expression of markers specific to the NP cell phenotype. In the mouse, studies have shown that the NP appears to consist entirely of notochordal cells which are retained into adulthood [50,51] and other studies with lineage-tracing techniques have also shown that all NP cells are originated from notochord [52,53]. For the reason that the mouse NP cell retains many features of the notochordally-derived cell, markers that relate to a notochordal cell phenotype may be used to test if mouse iPSCs can differentiate into mouse NP cells. This approach and interest forms the basis for this proposed study.

In prior studies, we reported that human umbilical cord-derived MSCs can differentiate into NP-like cells using a Matrigel-based (laminin-rich) culture system [33]. While the mechanisms regulating this cell differentiation remain largely unknown, the presence of the laminin ligand in the matrix and soft stiffness of the Matrigel substrate itself appear to match important characteristics of the native NP tissue for immature porcine NP. Culture of immature porcine NP cells under these conditions can maintain mainly notochordal-like NP cells, promote matrix biosynthesis, and maintain NP cell cluster morphology as well as NP unique marker expression (i.e. laminin 511 and specific integrin receptors) [8,33,54]. Herein, we perform studies using mouse iPSCs cultured in this similar laminin-rich environment to determine if these conditions can similarly promote expression of a unique notochordal-like NP cell phenotype. We first evaluated the expression of NP precursor markers in populations of mouse iPSCs cultured *in vitro* prior to differentiation or following spontaneous differentiation into embryoid bodies (EBs). We identified a subpopulation of CD24^+^ cells, indicative of NP precursor cells [13] in undifferentiated iPSCs. We then isolated these subpopulations of CD24^+^ iPSCs via magnetic activated cell sorting (MACS), to test the potential for this enriched CD24^+^ fraction to promote appropriate NP matrix regeneration in a laminin-rich culture system. Separately, unsorted iPSCs were studied for potential enhancement to the differentiation process of iPSCs towards an notochordal-like NP cell fate using select environmental conditions of low oxygen tension (hypoxia) [28] and notochordal cell-conditioned medium (NCCM) [31,32,55] under the same laminin-rich culture conditions.

## Materials and Methods

### iPSC generation and flow cytometry analysis

Using a lentiviral gene delivery system, primary mouse embryonic fibroblasts (MEFs, Millipore, Billerica, CA) were stably transduced with a poly-cystronic vector containing the inducible promoter Tet-On^TM^ (FU.tet.on. OSKM), which controlled expression of the four transcription factors: *Oct4, Sox2, Klf4* and *Myc* as reported in the previous study [45]. Briefly, following cell transduction with the lentiviral vector cells were cultured in stem cell culture medium containing DMEM / High Glucose (Life Technologies, Grand Island, NY), fetal bovine serum (15%, Atlanta Biologicals, Lawrenceville, GA), non-essential amino acids (1%, Life Technologies), sodium pyruvate (1%, Life Technologies), Glutamax-I (1%, Life Technologies), βMercaptoethanol (55µM, Life Technologies), gentamicin (25µg/ml, Life Technologies), valproic acid (0.5mM, Sigma, St. Louis, MO), leukemia inhibitory factor or LIF (103U/ml, ESGRO®, Millipore), and Doxycycline (10µg/ml, Sigma). Doxycycline was removed 7 days post induction of transcription factor expression and individual iPSC colonies were mechanically picked and expanded on a feeder layer of mitotically inactivated mouse embryonic fibroblasts (MEFs). Our prior work has shown that these iPSC colonies expressed proteins of pluripotency marker (OCT4, SSEA1 and NANOG) and were capable of differentiating into functional cardiomyocytes [45].

**Figure 1:**
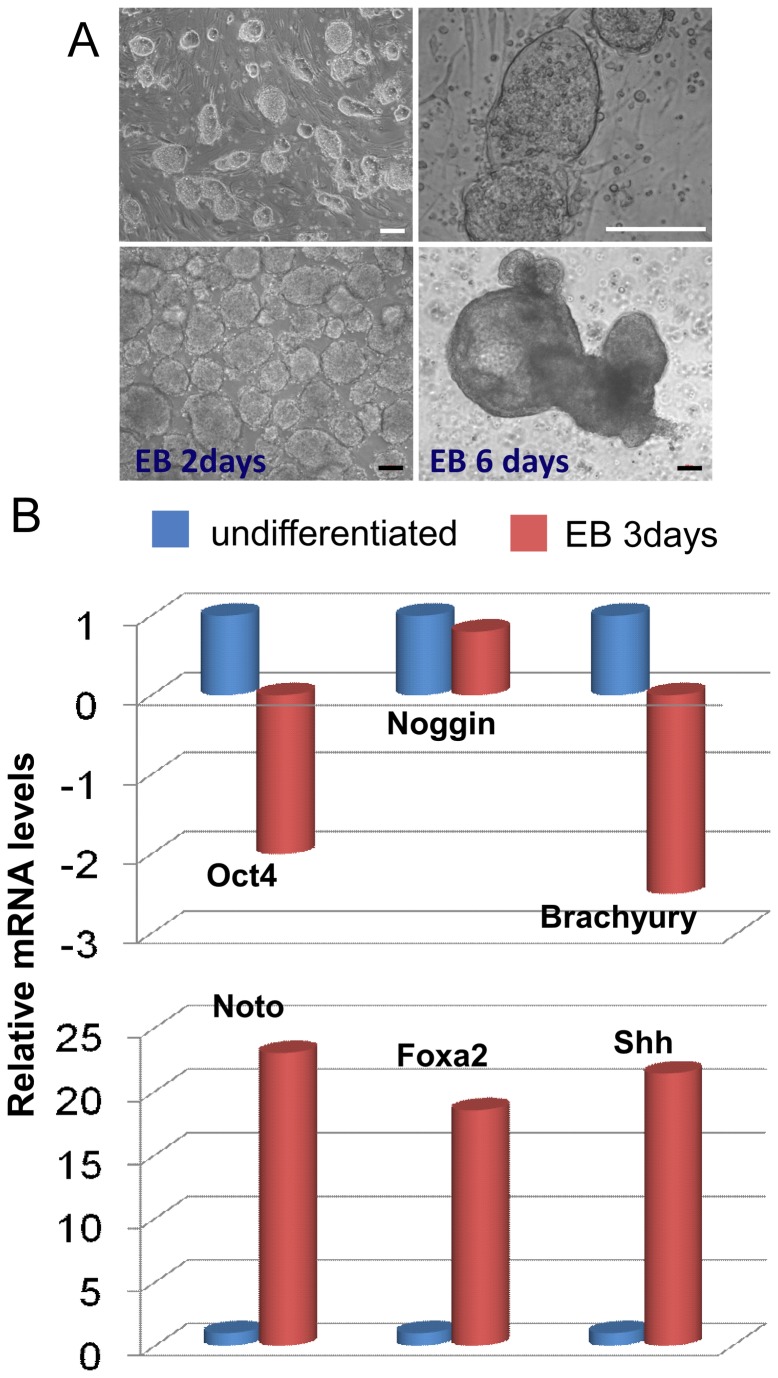
iPSC morphology and the expression notochordal-related transcriptional factors. (A) Undifferentiated iPSCs cultured on a feeder layer of mitotically inactivated mouse embryonic fibroblasts and formed colonies (top panels); iPSCs formed cell clusters of embryoid bodies (EBs, bottom panels) when promoted to undergo spontaneous differentiation in ultra-low attaching dishes (bars = 100 µm). (B) Relative mRNA levels for transcriptional factors (*Oct4, Noggin, Brachyury, Noto, Foxa2, Shh*) in EBs (3 days) normalized to undifferentiated iPSCs for each target gene. Data are averages of two replicates for cells collected from multiple EBs and undifferentiated culture.

For cell proliferation, undifferentiated iPSCs were seeded on a MEF feeder layer in proliferation medium (DMEM, 20% FBS and LIF, 1000 U/ml) and passaged twice (P2). For spontaneous differentiation, undifferentiated cells were allowed to form 3D embryoid bodies (EBs) in ultra-low attachment dishes (Corning, Corning, NY) with proliferation medium w/o LIF for 3 days (Figure 1). Gene expression for notochord-related transcription factors (*Noto, Foxa2, Shh, Brachyury, Noggin*) and the pluripotency marker *Oct4* was analyzed for both undifferentiated cells and cells in EBs via real-time RT-PCR (see below). In addition, the expression of MSC and NP-related biomarkers were also analyzed for both undifferentiated cells and cells in EBs by flow cytometry. Briefly, EBs were detached from the culture surface, and individual cells were dissociated using 0.025% trypsin/EDTA (Lonza, Walkersville, MD) and re-suspended in culture medium prior to antibody labeling. Cells (0.25~0.5x10^6^) were incubated with monoclonal antibodies against multiple cell markers (see Table 1) and appropriate isotype controls, followed by incubation with fluorescently labeled secondary antibodies. Cells were analyzed on an Accuri C6 flow cytometer (Accuri, Ann Arbor, MI) to determine the percentage of cells with positive surface proteins (% cells). Flow cytometry analysis was repeated for two sets of cell preparations. The average of values for percentage of positive cells was reported.

**Table 1:** Cell markers for NP phenotype analysis.

**Antibody**	**Vendor**	**Host/type**	**Isotype control**	**Application**
CD24	BD Biosciences	Rt/mono	Rt IgG2b	FC and MACS
CD54	BD Biosciences	Ha/mono	Ha IgG1	FC
Integrin α3 (CD49c)	R&D Systems	Go/poly	N/A	FC
Integrin α5 (CD49e)	BD Biosciences	Rt/mono	Rt IgG2a	FC
Integrin α6 (CD49f)	BD Biosciences	Rt/mono	Rt IgG2a	IHC and FC
Integrin β1 (CD29)	BD Biosciences	Ha/mono	Ha IgM	FC
Integrin β4 (CD104)	Serotec	Rt/mono	Rt IgG2a	FC
CD31	BD Biosciences	Rt/mono	Rt IgG2a	FC
CD34	BD Biosciences	Rt/mono	Rt IgG2a	FC
CD45	Serotec	Rt/mono	Ra IgG2b	FC
CD90	Serotec	Ms/mono	Ms IgG1	FC
Tie	Biolegend	Rt/mono	Rt IgG1, k	FC
GD2	BD Biosciences	Ms/mono	Ms IgG2a	FC
Type II collagen	DSHB	Ms/mono	Ms Igg	IHC
Laminin α5	Santa Cruz	Rb/poly	NA	IHC
Cytokeratin5/8	Santa Cruz	Ms/mono	Ms IgG1	IHC
Vimentin	Santa Cruz	Ms/mono	Ms IgG1	IHC

Go: goat, Ha: hamster, Hu: human, Ms: mouse, Rb: rabbit, Rt: rat, IHC: immunohistochemistry, FC: flow cytometryNA: not available; mono: monoclonal; poly: polyclonal.

### Enrichment of CD24^+^ cell population from undifferentiated iPSCs by MACS

Freshly trypsinized mouse iPSCs (10x10^6^ cells) were partitioned into 1.5 mL Eppendorf tubes, and suspended in incubation buffer (1X DPBS without Ca^2+^ and Mg^2+^, 0.5% BSA) and rat anti-mouse CD24-FITC (BD Biosciences, San Diego, CA) for an hour. For each sample, cells (0.5x10^6^ cells) were mixed with incubation buffer and the isotype control (rat IgG2b-FITC, AbD Serotec, Kidlington, UK) for the same duration. After centrifugation (600 rcf, 6 minutes) and supernatant removal, iPSCs were washed and re-suspended in labeling buffer (1X DPBS without Ca^2+^ and Mg^2+^, 0.5% BSA, 2 mM EDTA) and anti-FITC microbeads (Miltenyi Biotec, Bergisch Gladbach, Germany) for 30 minutes.

**Figure 2:**
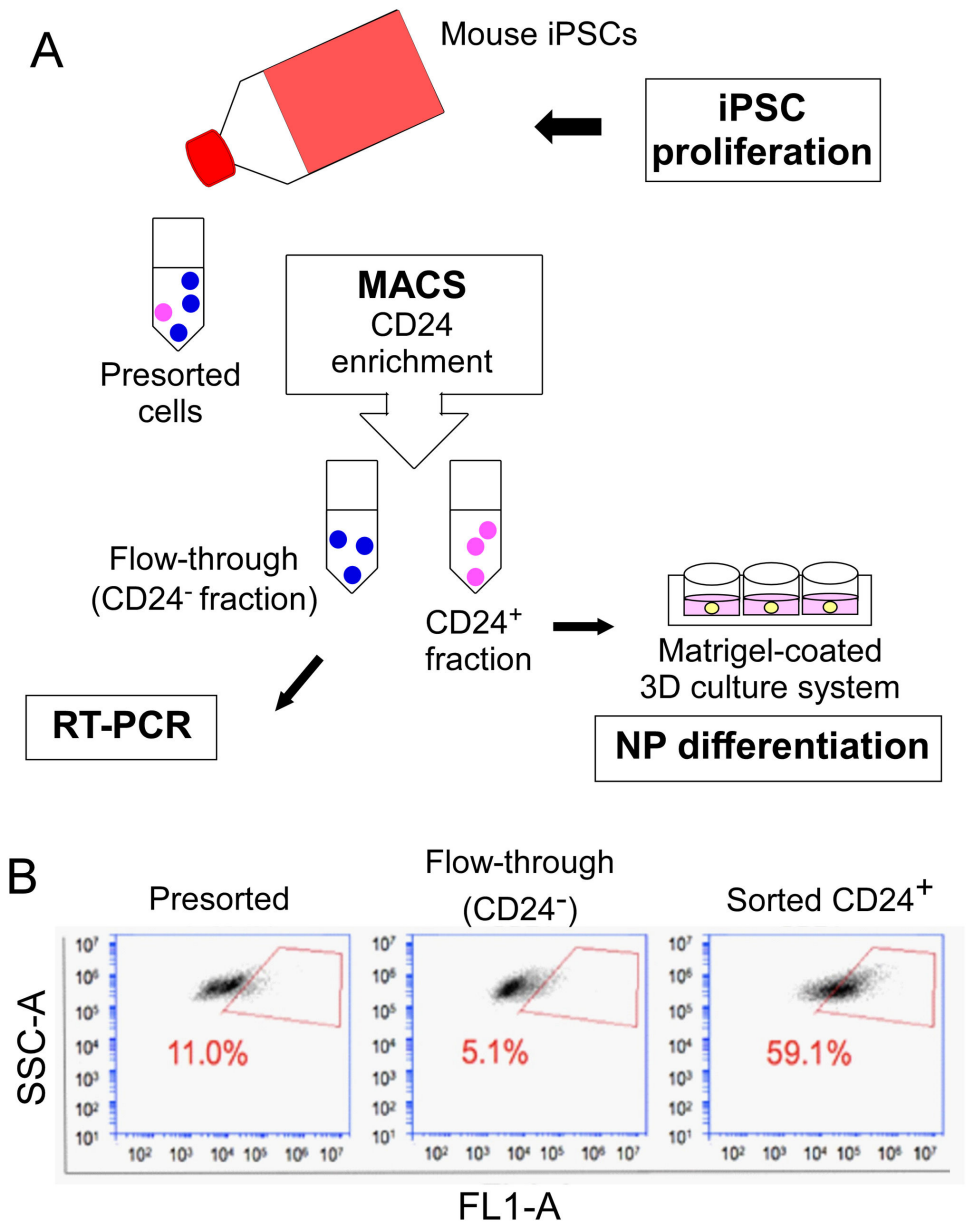
Enrichment of CD24^+^ cell population from undifferentiated iPSCs via magnetic activated cell sorting (MACS). (A) General experimental overview. (B) Flow cytometry analysis for the expression of CD24 in presorted, “flow-through” and sorted cell fractions. Undifferentiated iPSCs incubated with CD24 antibody and magnetic beads were passed through a MACS column, yielding a CD24^+^ fraction of cells; in the “flow-through” fraction, ~ 5.1% of total cells on average were found to express CD24, which was less than that in the presorted fraction. The gated readouts represent the percentage of cells expressed CD24 in presorted cells and sorted fractions from one sorting experiment.

The MiniMACS^TM^ kit (Miltenyi Biotec) was used to sort for CD24-positive cells using the MiniMACS^TM^ separator and MACS magnetic column. This column was rinsed with separation buffer before loading of the iPSC suspension. Three flushes with separation buffer (1X DPBS without Ca^2+^ and Mg^2+^, 0.5% BSA, 2 mM EDTA, degassed) resulted in the collection of a “flow-through” volume consisting of unlabeled cells (CD24 negative, CD24^-^). Meanwhile, magnetically labeled cells were retained on the MACS column and subsequently eluted via a plunger (CD24 positive, CD24^+^). The small amount of cells (~10,000) from both CD24^+^ fractions and the “flow-through” fraction (CD24^-^), along with a presorted sample, were analyzed with a flow cytometer (Accuri C6) for the percentage of cells expressing CD24. In general, this CD24 sorting protocol was able to provide an approximately five-fold enrichment for the percentage of CD24 expressing cells (59-85%) in the sorted fraction as compared to presorted cells (12-17%). Generally, the amount of CD24-expressing cells in the “flow-through” fraction were found to be less than 5% of total cells demonstrating excellent capture of CD24^+^ cells within the magnetic column (Figure 2). A minimum of six independent sorting experiments were performed in order to collect enough both CD24^-^ cells in “flow-through” and sorted CD24^+^ cells for gene expression analysis and for 3D culture of CD24^+^ enriched cell populations.

### RNA Isolation and Realtime RT-PCR

Total RNA was extracted from cells in the following groups (Group I: undifferentiated iPSCs and cells in EBs for 3 days, n=2; Group II, unsorted iPSCs, CD24^-^ cells in “flow-through” and sorted CD24^+^ cells immediately after cell sorting, n=3) with the RNAeasy kit plus DNase I digestion (Qiagen, Valencia, CA, USA), as previously described [56]. For quantitation of mRNA, two human-specific PCR primers and one fluorescently labeled intron-spinning probe were used for each target gene (Applied Biosystems, Foster City, CA, Table 2), with amplification conditions used as previously described [56]. Relative gene expression differences were quantified between the control (undifferentiated or pre-sorted iPSCs) and treatment group (EB or CD24^-^ or CD24^+^ iPSCs) using the comparative C_t_ method with β2-microglobulin (*B2m*) as the internal control. The expression of this housekeeping gene was stable during differentiation of iPSCs according to our preliminary analysis. The relative mRNA level of undifferentiated cells or presorted cells was set as the calibrator (value=1) and EB cells or sorted CD24^+^ cells were normalized based on this value. Duplicate PCR reactions were performed for each target gene and the internal control for one RNA sample. Statistical analyses were performed to detect a difference in ΔC_t_ (C_t_ of target -C_t_ of *B2m*) values between presorted and sorted CD24^+^ or CD24^-^ samples for each target gene using a one-factor ANOVA (StatView, SAS Institute, Cary, NC). Fold-differences in relative mRNA levels (2^-∆∆Ct^) between control (presorted cells) and sorted samples (CD24^+^ or CD24^-^) were reported if greater than or equal to 2, and where ANOVA detected a difference at p<0.01 [56].

**Table 2:** Probes and primers of notochordal-cell markers analyzed by real-time RT-PCR (Applied Biosystems).

**Target**	**PCR probe/primers Order number**
*Noto*	Mm02345142_m1
*Foxa2*	Mm01976556_s1
*Noggin*	Mm01297833_s1
*Brachyury (T*)	Mm01318252_m1
*Shh*	Mm00436528_m1
*Oct4*	Mm03053917_g1
*β-2-microglobulin (B2m*)	Mm00437762_m1

### Differentiation experiment I: an enriched CD24^+^ iPSC population cultured *in vitro*

As outlined in Figure 2A, both CD24^-^ cells in “flow through” and enriched CD24^+^ iPSCs via MACS were allowed to differentiate in a laminin-rich culture substrate previously shown to promote the formation of a three-dimensional cell clusters. Briefly, 60 µL of Matrigel^TM^ (BD Biosciences, San Jose, CA) was coated onto 6.5 mm diameter Costar Transwell inserts (Corning), and let set at 37°C for 2 hours. Immediately after sorting, the CD24^-^ and CD24^+^ fractions of iPSCs were seeded onto the Matrigel-coated surfaces at 1x10^6^ cells per insert (n=3) arranged in a 24-well plate. Both cell types have been observed to cluster into large cell pellets (or 3D construct) within 24 hours at these conditions [54] that are stable over long periods of culture. Cell constructs were cultured under normoxic conditions (21% O_2_) for up to 28 days in NP differentiation medium: DMEM/ F-12 with 15 mM HEPES, L-glutamine, and pyridoxine hydrochloride (1:1, v/v; Life Technologies), with additional L-ascorbic acid-2-phosphate (sterilized using a 0.22 µm filter; Sigma), non-essential amino acids, insulin transferrin-selenium (ITS) and penicillin-streptomycin (all from Life Technologies). Medium both outside and inside the inserts was changed biweekly for the duration of cell culture.

### Differentiation experiment II: iPSCs cultured under hypoxic conditions

Transwell inserts were coated with Matrigel^TM^ as described above. When nearly confluent, tissue culture flasks of proliferated iPSCs were transferred to a low-oxygen humidified environment (2% O_2_, 37°C) for a 24-hour period. Following this protocol for hypoxic conditioning, cells were gently detached using 0.025% trypsin/EDTA (Lonza, Walkersville, MD) and seeded onto Matrigel-coated Transwells (n=3) as described above. They were then cultured in either equilibrated NP differentiation medium (see above), or in equilibrated media collected from immature NP tissue explant culture, termed notochordal cell conditioned medium (NCCM). For generation of NCCM media, immature porcine NP tissues containing largely notochordal-like cells were incubated in DMEM-based culture media under hypoxic conditions for 4 days as described by Purmessur and co-workers [32]. At the end of the incubation, the conditioned media was collected, concentrated, and stored at -80°C until used as a supplement to iPSC culture. For iPSCs cultured under these conditions, medium were changed twice per week for up to 7, 14, 21 and 28 days in both outside and inside the Transwell inserts. Similarly, iPSCs also have been observed to cluster into large cell pellets (or 3D construct) within 24 hours under hypoxia and NCCM culture conditions that are stable over long periods of culture.

### Histological evaluation for cell morphology and proteoglycan composition

At the end of each time point, samples of cultured cell constructs were embedded in OCT medium (Sakura Finetek, Torrance, CA) and immediately flash-frozen in liquid nitrogen. As a native IVD tissue control, lumbar IVDs were harvested from a 1month-old mouse, then embedded and flash-frozen. They were subsequently stored at -80°C until cryosectioning. Frozen sections (7 µm) of IVD tissue and cell construct were then fixed in 10% neutral buffered formalin (Azer Scientific, Morgantown, PA) for 10 minutes, and washed in 1% lithium carbonate solution (Mallinckrodt Chemicals, Phillipsburg, NJ) and stained with 0.5% safranin-O solution (Sigma, St. Louis, MO) for 60 seconds. Samples were rinsed with distilled water and counterstained with Mayer’s Hematoxylin (Sigma) to visualize individual cells. After serial steps of dehydration, sections were then mounted with Permount (Fisher Scientific, Pittsburg, PA) and visualized with light microscopy for intensity of glycosaminoglycan staining.

### Immunohistochemical staining for type II collagen

Frozen sections of IVD tissue and cell construct were processed for immunohistochemical labeling of type II collagen (HistoMouse-Plus kit, Invitrogen, Camarillo, CA) as described previously [57]. Following peroxide-blocking, sections were digested with pepsin (Digest-All 3, Life Technologies) for 10 minutes at 37°C. This enzyme helped expose the type II collagen epitope recognized by the mouse monoclonal anti-chicken antibody (II-II6B3, Developmental Studies Hybridoma Bank, Iowa City, IA). Negative control sections were processed in parallel with mouse IgG isotype control (Millipore) instead of the primary antibody.

### Immunohistochemical staining for NP-markers

Frozen sections of IVD tissue and cell construct were fixed in 4% formaldehyde (10 minutes at room temperature) prior to labeling with antibodies detecting NP markers; for labeling of the integrin α6 subunit, tissue sections were fixed in acetone (10 min at -20°C). All fixed tissue sections were incubated with a blocking solution (3.75% BSA/5% goat serum, Invitrogen) for 30 minutes, and then incubated for 2 hours with one of the following antibodies: monoclonal or polyclonal anti-human laminin α5 chain, vimentin, cytokeratin5/8, integrin subunit α6 (Table 1). Sections were washed twice in PBS and incubated with appropriate secondary antibodies (AlexaFluor 488 or 633 secondary antibodies, Molecular Probes, Eugene, OR) for 30 minutes in blocking solution. Negative control sections were incubated with appropriate isotype IgG controls (Table 1) instead of primary antibody, or with secondary antibody alone as a negative control (polyclonal antibodies). All sections were counterstained with propidium iodide (0.2 mg/mL, Sigma) to label cell nuclei, and imaged using confocal laser scanning microscopy (Zeiss LSM 510; 20X NA 0.5 and 63X water immersion NA 1.2 objectives; Carl Zeiss, Thornwood, NY).

### sGAG content

Differentiated iPSC production of sulfated glycosaminoglycans (sGAGs) was analyzed using the dimethylmethylene blue (DMMB) spectrophotometric method [58]. All samples of cell constructs were digested in papain solution (300 µg/mL in PBS with 5mM EDTA and 5mM cysteine, Sigma; 65°C for 16 hours) and the sample digest was stored at -20°C. sGAG content in sample digest (40 µl) was measured by mixing with DMMB dye solution (125 µl, 21µg/ml, pH = 3; 2 replicates per sample) in a 96-well assay plate and measuring absorbance (535nm) on a plate reader (Tecan Genios, Mannendorf, Switzerland). sGAG concentrations were calculated from absorbance using a standard curve prepared from commercial chondroitin-4-sulfate (Sigma). Total sGAG per sample was normalized to DNA content similarly measured in sample digests (Quant-iT PicoGreen dsDNA Kit, Invitrogen). Differences in sGAG content (sGAG/DNA) amongst control and treatment groups at each time point were evaluated via two-factor ANOVA, followed by Turkey’s post-hoc analysis at a significance level of 0.05 (n=4).

### Sircol collagen assay

Papain-digests were also analyzed for collagen content samples using the Sircol Collagen Assay Kit (Biocolor; Northern Ireland, UK). Digested samples (40µl) were incubated with the Sircol Dye reagent for 30 minutes with gentle mixing, then centrifuged (10,000 g for 10 minutes) to collect the collagen-bound dye. Following supernatant removal, the pellet was re-suspended in alkali reagent and transferred to a 96-well assay plate for measuring absorbance (535nm, Tecan Genios) with total collagen content calculated using a standard curve. A negative control with only papain solution (40µl) was tested to ensure that papain did not interfere with absorbance reading of collagen assay. Two duplicate wells were measured for each sample, and each collagen content value was normalized to DNA content (as described above) for each sample. Differences in total collagen production (collagen/DNA) amongst control and treatment groups at each time point were evaluated via two-factor ANOVA, followed by Turkey’s post-hoc analysis at a significant level of 0.05 (n=3).

## Results

### iPSC derivation and characterization

Mouse embryonic fibroblasts were epigenetically reprogrammed into induced pluripotent stem cells following their transduction with an inducible expression polycystronic lentiviral vector allowing the controlled expression of transcription factors *Oct4, Sox2, Klf4*, and *Myc* [45]. Previous results confirmed successful reprogramming of mouse embryonic fibroblasts and the generated iPS cells expressed proteins of pluripotency marker OCT4 (nuclear localization), NANOG (nuclear localization), and SSEA1 (cell surface localization) as determined by immunofluorescence [45]. In the current study, the iPSCs proliferated and formed colonies on mitotically inactivated MEFs feeder layers (Figure 1A, top). When cultured in ultra-low attachment dishes without LIF for spontaneous differentiation, iPSCs formed EB-like cell clusters (Figure 1A, bottom) similar to embryonic stem cells.

Undifferentiated iPSCs and EBs (at 3days) expressed many integrins (α3, α5, α6 and β1 subunits) and other NP markers (CD24 and CD54) on their cell surface (Table 3). An elevated percentage of cells expressed CD24 (NP cell marker), CD90 (mesenchymal stem cell marker) and CD31 (endothelia cell marker) in EBs as compared to undifferentiated cells (Table 3). Interestingly, about 10% of undifferentiated iPSCs expressed Tie2, a recently identified marker for NP progenitor cells [13], although its expression was declined in EBs (3%). It was noted that the integrin β4 subunit, CD45, CD34 and GD2 (another marker of NP progenitor cells [13]) were not expressed by undifferentiated cells or by cells in EBs. In addition, EBs expressed significant higher levels of mRNA for *Noto, Foxa2* and *Shh* (notochordal genes) as compared to undifferentiated cells (Figure 1B). Relative gene expression levels for pluripotent marker *Oct4* or nascent mesodermal marker *Brachyury* decreased in EBs (Figure 1B).

**Table 3:** NP and MSC associated marker expression in mouse iPSCs determined via flow cytometry analysis.

**Marker**	**% Positive Cells Undifferentiated**		**% Positive Cells EB 3 days**	**Known cell associations**
CD24		17	28	NP
CD54		54	35	MSC, NP
CD49c (ITGα3)		22	9	MSC, NP
CD49e (ITGα5)		32	27	MSC
CD49f (ITGα6)		27	27	MSC, NP
CD29 (ITGβ1)		81	85	MSC
CD104 (ITGβ4)		0	3	NP
Tie		10	3	NP progenitor
GD2		0	0	NP progenitor
CD90		0	15	MSC
CD31		3	20	Endothelial
CD45		0	2	Leukocyte
CD34		0	1	Hematopoietic

Values represent an average percentage of cells expressing the markers (n = 2 samples, 10^4^ cells per sample); ITG: integrin.

### An enriched CD24^+^ fraction of iPSCs

**Figure 3:**
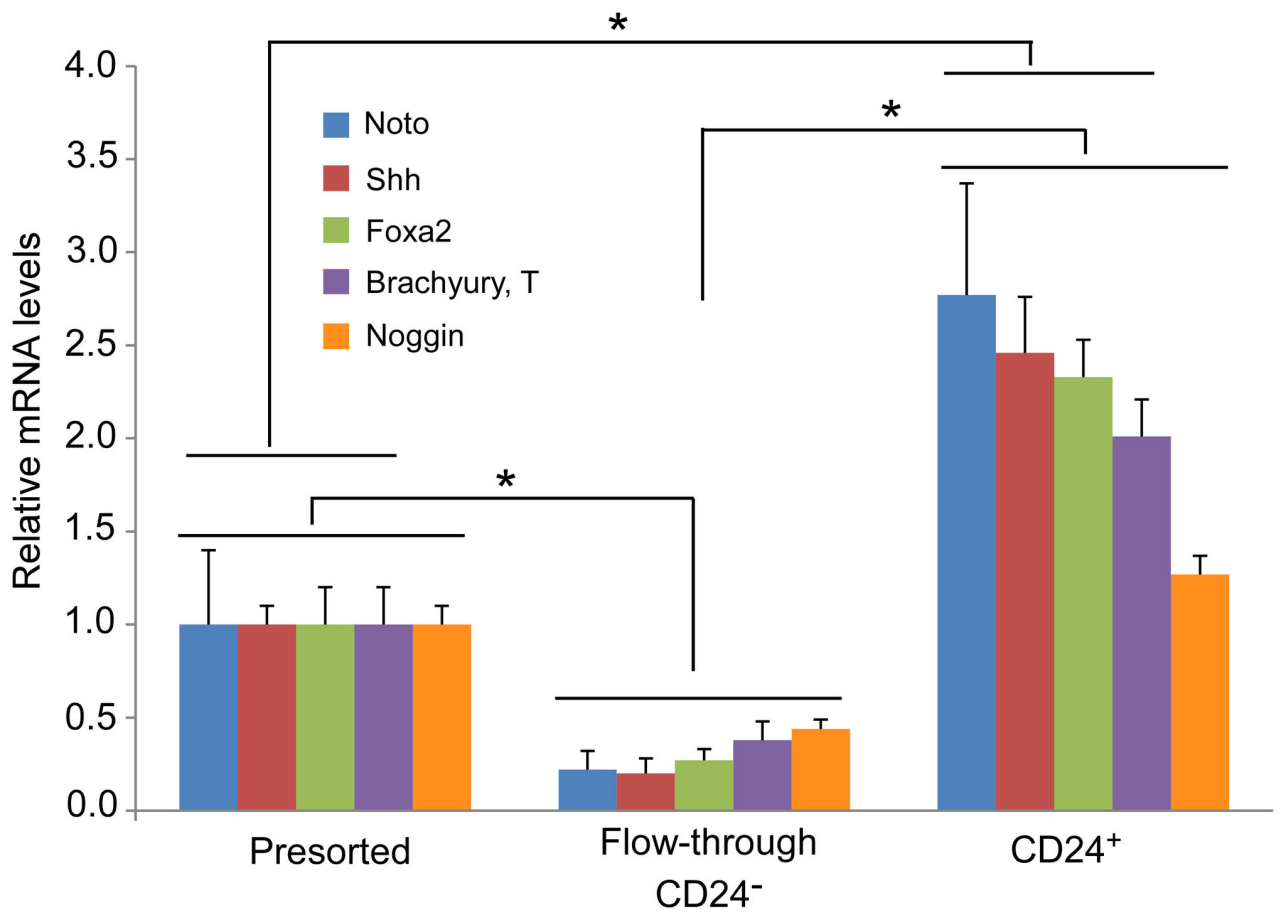
Relative mRNA levels in undifferentiated, presorted iPSC populations, “flow-through” and sorted CD24^+^ fractions for notochordal markers (*Noto, Shh, Foxa2, Brachyury* and *Noggin*). Data are expressed as fold-difference (2^-ΔΔCt^, n=3 independent sorting experiments, mean+SE) between sorted CD24^+^ or “flow-through” and presorted samples. The relative mRNA level in presorted cells was set as the calibrator (value=1) for normalizing the relative mRNA levels in the CD24^+^ cell fraction and “flow-through” fraction (* p<0.01, ANOVA: significant fold-difference detected between presorted cells and CD24^+^ cells or CD24^-^ cells in “flow-through”, and between CD24^-^ cells in “flow-though” and CD24^+^ cells). Differences were detected in all targets except for Noggin between unsorted cells and sorted CD24^+^ cells.

CD24^+^ cell fractions obtained from MACS as well as presorted cells and CD24^-^ cells in the “flow-through” fraction were quantified by flow cytometry and analyzed for gene expression of notochordal related transcription factors. Following MACS, CD24^+^ iPSCs exhibited a 2 to 5-fold increase in mRNA for *Noto, Shh, Foxa2* and *Brachyury*, as compared the presorted cells (Figure 3). Differences in mRNA for *Noggin* were not detected following CD24^+^ selection. In contrast, mRNA for *Noto, Shh, Foxa2*, *Brachyury* and *Noggin* was decreased between 2 and 5-fold in CD24^-^ cells of the “flow-through” fraction, as compared to the presorted cells (Figure 3). These findings corroborate the identification of CD24^+^ as a marker of the NP precursor cell that is associated with expression of a set of signaling molecules and transcription factors (*Shh, Foxa2, Noto* etc.) closely identified within notochord [53,59-61].

### Differentiation of enriched CD24^+^ iPSCs *in vitro*

**Figure 4:**
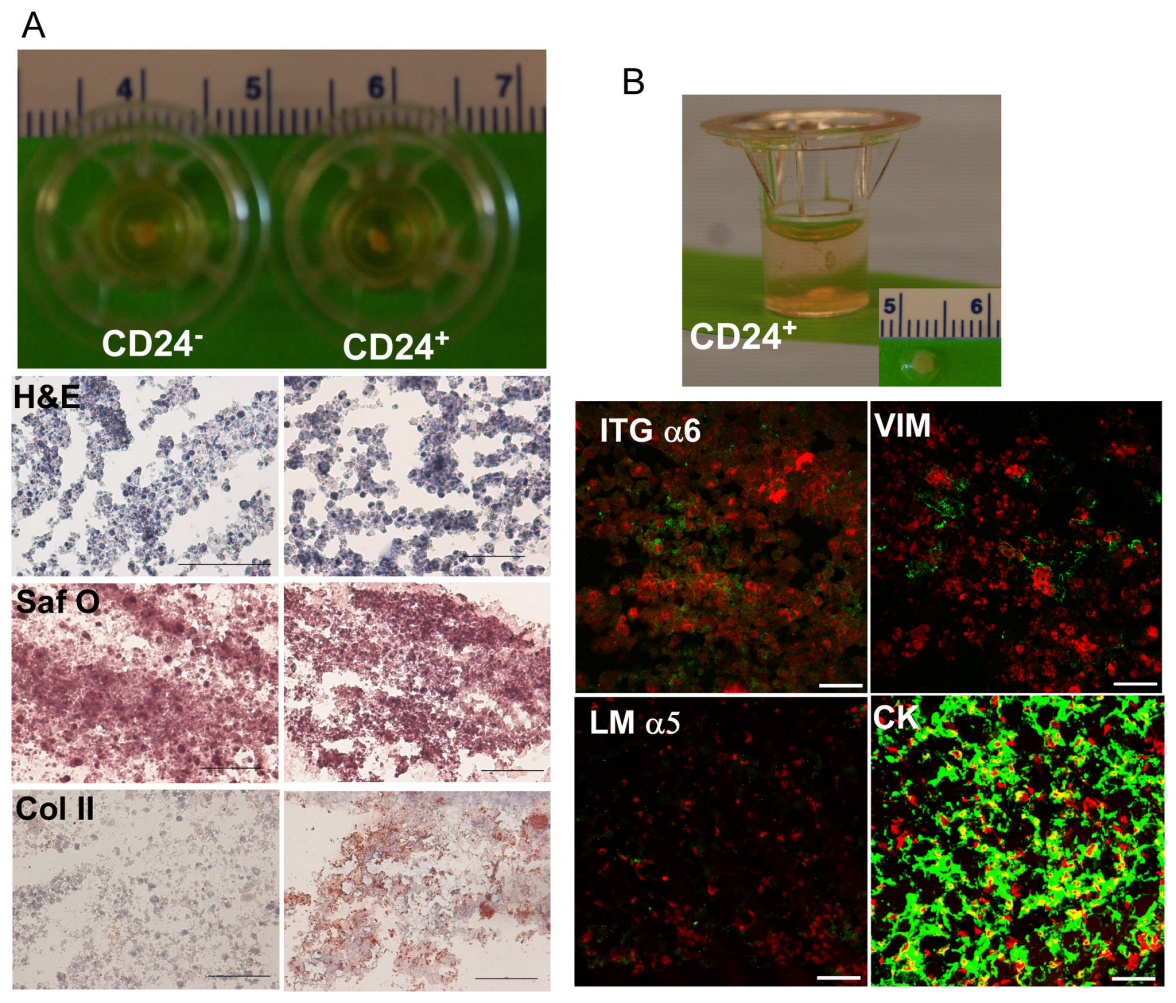
Differentiation of enriched CD24^+^ iPSCs in a 3D culture system *in vitro*. (A) Cell constructs form both CD24^-^ and CD24^+^ fractions extracted from Transwells at the end of culture (day 28) and representative images of histology and immunostaining revealed cell morphology (H and E) and matrix accumulation (Saf O for glycosaminoglycan and type II collagen, Col II; Scale bar: 50 µm). (B) CD24^+^ cell construct and representative images for expression of NP-associated markers by immunostaining (integrin α6, ITGα6; laminin α5, LM α5; vimentin, VIM and cytokeratin 5/8, CK) in cell constructs at day 28. Scale bar: 20 µm).

Both CD24^-^ and CD24^+^ cells formed large cell clusters upon the laminin-rich (Matrigel) culture system within 24 hours, then remained stable over time during culture. When cultured on this soft gel substrate at 20% O_2_ and with differentiation media, these cells formed cell clusters (Figure 4A, H& E staining), expressed glycosaminoglycan (sGAG) (Figure 4A, Safranin-O staining), and maintained a gelatinous morphology (Figure 4 A& B top) reminiscent of immature NP cells *in situ* for the duration of culture [54]. Interestingly, only tissue sections from CD24^+^ cell constructs showed abundant expression of type II collagen (immunostaining, Figure 4A) which was not detected in tissue sections of CD24^-^ cells. In addition, we also detected laminin α5 expression (immunostaining), although at very low levels in CD24^+^ cell constructs (Figure 4B). Similarly, CD24^+^ cells within these constructs expressed some markers of the immature NP cell phenotype including integrin α6 (known to interact with laminins), vimentin and cytokeratin 5/8 (Figure 4B), while some positive expression for these NP-associated markers were also detected in CD24^-^ cells (data not shown).

### Differentiation of iPSCs under hypoxic conditions

**Figure 5:**
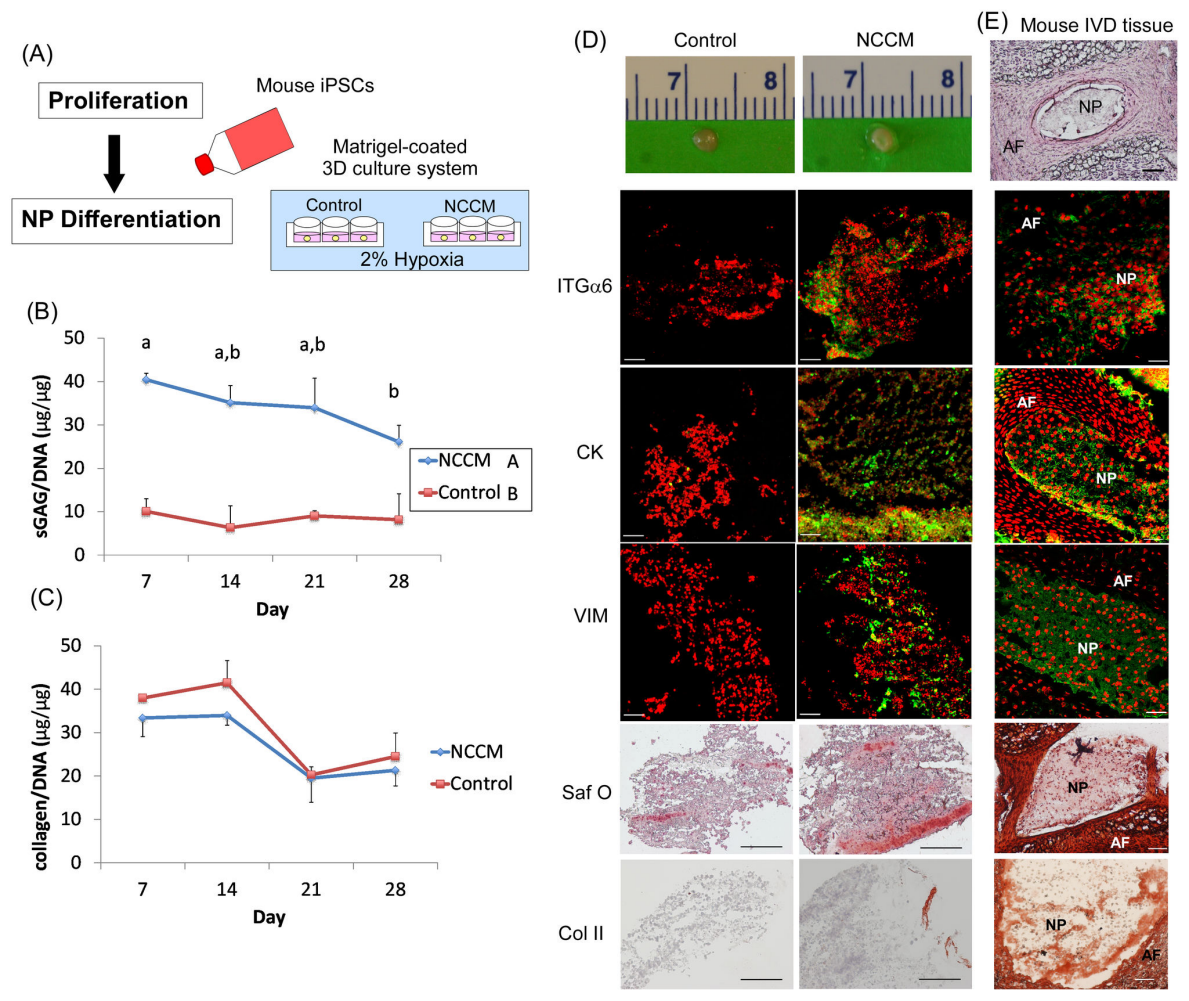
Differentiation of iPSCs under hypoxic conditions *in vitro*. (A) General experimental overview. (B) Sulfated-glycosaminoglycan content normalized to DNA content (sGAG/DNA) in cell constructs (results shown as mean+SD, n=3 samples). Significant effects of treatment and time were detected using a two-way ANOVA and Tukey’s HSD (p<0.05). Treatment and time points denoted by different letters were found to be significantly different: uppercase letters for treatment; lowercase letters for time. (C) Total collagen content normalized to DNA (collagen/DNA) in cell constructs (results shown as mean+SD or –SD, n=3 samples). No significant effects of treatment on collagen production were detected via two-way ANOVA and Tukey’s HSD (p>0.05). (D) Cell constructs extracted from Transwelsl at the end of the culture (day 28) and representative images of histology and immunostaining revealed matrix accumulation (Saf O for glycosaminoglycan and type II collagen, Col II; Scale bar: 100 µm) and expression of NP-associated markers (vimentin, VIM; cytokeratin 5/8, CK and integrin α6, ITGα6) in cell constructs at days 28. Scale bar: 50 µm. NCCM: notochordal cell conditioned medium. (E) Representative images of histology (H and E) and immunostaining for above mentioned NP-associated markers and matrix proteins in the immature mouse IVD (1 month-old) tissue control (AF: anulus fibrosus; NP: nucleus pulposus).

Studies were also undertaken to determine if unsorted iPSCs could be promoted to differentiate into NP-like cells under hypoxic culture conditions (2% O_2_), a condition commonly used for NP cell culture and believed to mimic the in situ oxygen tension [28]. In addition, comparison studies were performed to determine if this same population of iPSCs could be promoted to differentiate into NP-like cells when cultured with factors secreted by notochordal cell-containing NP tissue, or NCCM (Figure 5A). When cultured on top of the laminin-rich (Matrigel) substrate, iPSCs formed large cell clusters that were stable over time during culture (Figure 5D top) and produced significant amounts of sGAG and collagen (Figure 5B & C) under hypoxic conditions, similar to that measured when using the CD24^+^ cell fraction in culture under normoxic conditions. The constructs cultured in NCCM produced significantly higher levels of sGAG at all times, reflecting the higher composition of anabolic growth factors in NCCM (Figure 5B). In contrast, few differences were noted in collagen composition between the control and the NCCM (Figure 5C). A prior study has shown that anabolic factors in media conditioned by culture of anulus fibrosus cells (immature porcine) produced equivalent levels of biosynthesis in de-differentiated cells as did NCCM [55], so the specificity of the anabolic factors to an NP cell source cannot be inferred.

When iPSC cell constructs were cultured under hypoxic conditions and with NCCM supplementation, some cells within these constructs expressed several markers of the immature NP cell phenotype, such as vimentin, cytokeratin 5/8 and integrin a6 subunit, as compared to the control (immunostaining, Figure 5D). Similarly, NCCM supplementation also enhanced matrix accumulation as indicated by Safranin-O staining for sGAG and immunostaining for type II collagen from some parts of cells (Figure 5D). In contrast to the CD24^+^ cell fraction in culture under normoxic conditions, expression of laminin α5 in all cell constructs under hypoxic conditions was not detected (data not shown). As a positive control, NP-associated markers (integrin α6, cytokertin 5/8 and vimetin) were also found to be highly expressed in NP cells but not in AF cells of immature mouse IVDs (Figure 5E). Both type II collagen and Safranin-O staining were clearly stained positive in this immature mouse NP tissue (Figure 5E).

## Discussion

The results of this study demonstrate that a laminin-rich culture environment may promote the differentiation of iPSCs or enriched CD24^+^ iPSCs into a cell type exhibiting some NP-like markers and immature NP morphology. ECM formation (proteoglycans, type II collagen and laminin 511 in differentiated CD24^+^ iPSCs) and the presence of several NP markers (integrin α6 subunit, vimentin and cytokeratin 5/8) were detected in the differentiated cells. These expression patterns of NP-like markers and clustering morphology were similar to those detected in immature NP tissue *in situ* (Figure 5E), primary NP cells and human umbilical cord-derived MSCs cultured under a similar laminin-rich environment in our previous studies [33,54]. While no complete set of markers has yet been established for NP cells, findings of this study suggest that iPSCs may be induced to differentiate into NP-like cells as demonstrated by the presence of multiple NP-associated markers. This is the first study in which iPSCs have been shown to differentiate into NP-like tissue, capable of synthesizing type II collagen and sGAG, as well as express various markers distinct to NP cell phenotype as confirmed in native mouse NP tissue. The CD24^+^ cell population and undifferentiated iPSCs cultured in NCCM exhibited features characteristic of NP-like tissue, including integrin α6, and the cell markers cytokeratins 5/8 and vimentin. Together with the detection of type II collagen and sGAG, these studies show accumulation of an NP-like matrix and retention of an NP-cell phenotype that has not been demonstrated in other MSC studies.

*In vitro* culture upon a Matrigel substrate was chosen here for its demonstrated ability to promote NP cell attachment [8], with the proposed mechanism that laminins within the Matrigel may preferentially promote attachment of iPSCs to form NP cells. It is known that immature NP cells reside in an unique ECM environment rich in specific laminin isoforms (LM511 and LM322) and that they express high levels of laminin-binding receptors (i.e. integrin α6 subunit) as compared to the surrounding AF cells [6-8]. Previous studies have shown that immature NP primary cells and human umbilical cord-derived MSCs will maintain a clustered morphology when cultured upon this laminin-containing Matrigel substrate [33,54], that may work to promote maintenance of the NP cell phenotype. Thus, recreation of this feature of the immature NP ECM environment, along with the soft stiffness of Matrigel substrate that matches that of the native NP [54], may have contributed to the directed differentiation of iPSCs here. Previously, laminin-containing substrates were found to enhance adult MSC differentiation to adipocytes [50] and human ESC differentiation into neurons [62], although with defined culture media that included lineage-specific growth factors or other inducers for adipogenesis and neural differentiation. A recent study of finding a population of multipotent progenitors from NP tissue made use of methylcellulose as a substrate [13], which has mechanical properties that match that of the Matrigel used here. Still, this prior work made use of serum supplementation but not laminin ligand presentation such that the culture microenvironment was quite distinct from that used here. We can conclude that the interaction of multiple factors that include ECM proteins and cell-ligand engagement, as well as growth factor supplementation and substrate stiffness, appear to be important for promoting iPSC differentiation into NP-like cells *in vitro* in our current study.

Directed differentiation for enriched CD24^+^ iPSCs was done without any growth factor supplementation, as is commonly applied for chondrogenesis with use of ESCs or iPSCs. Clearly, the finding of collagen II only detected in CD24^+^ cells indicated that CD24^+^ cells were differentiated into cells with more chondrogenic potential than CD24^-^ cells. In addition, differentiation of unsorted iPSCs was tested under both hypoxic conditions and with NCCM supplementation. In fact, NCCM generated from native NP tissues may be expected to contain many soluble factors including TGFβ, BMPs and other growth factors which commonly present in IVD tissues [63], such that culture with NCCM would have been expected to promote a more chondrogenic phenotype. In one study, connective tissue growth factor has been identified in NCCM and has been shown to act as an anabolic factor, promoting aggrecan gene expression in primary NP cells [64]. Similarly, NCCM has been shown to promote MSC differentiation into NP-like cells and to enhance gene expression of matrix proteins in MSCs, aged human NP cells and porcine IVD cells [31,32,55,65]. Indeed, we saw that NCCM had a significant effect in mediating iPSC differentiation and promoting the accumulation of more NP-like matrix. However, the enriched CD24^+^ cells were also capable of synthesizing NP-like matrix, with only the culture conditions (without growth factors) being the dominant driver of differentiation and matrix production. Supplementation with NCCM seemed to improve matrix synthesis, notably the accumulation of sGAG that is considered important for regenerating the NP – a structure abundant in proteoglycans. These findings for NCCM-cultured iPSC constructs, which were already at levels reported for porcine NP cells cultured on Matrigel in our previous study [54]. Total collagen synthesis did not depend on treatment type, however, but varied significantly over time (days 7 and 14 > days 21 and 28). It is suspected that an appreciable amount of collagen may have been released into the surrounding culture environment during the differentiation process. Validation of this will involve collecting culture media at various time points and repeating biochemical quantification.

Previous studies have demonstrated that distinct cell populations (i.e., ESC-derived pancreatic progenitors, epithelial cells-drived MSCs) may be isolated via CD24 sorting [66,67]. CD24 has also been identified as a marker of NP cells [11] and of progenitor cells in NP [13]. The current study showed that mouse iPSCs express CD24, albeit at low cell numbers. These observations provided the impetus for MACS to obtain an enriched CD24^+^ fraction of mouse iPSCs. CD24 was corroborated as an immature NP cell marker through the identified upregulation of mRNA for *Noto, Shh, Foxa2* and *Brachyury* notochordal markers indicating a potential NP cell lineage in this enriched CD24^+^ fraction of iPSCs as compared to CD24^-^ cells. CD24^+^ selection alone proved sufficient to insure accumulation of matrix with abundant type II collagen although NP-associated markers (integrin α6, laminin, and cytokeratins 5/8 and vimentin) were also detected in CD24^-^ cells. While some similar expression patterns were observed for the unsorted iPSCs cultured in NCCM, this strategy of sorting cells prior to directed differentiation can have advantages over the use of stem cells directly, that can enable differentiation into a specific lineage and also reduced tumor genic potential. A previous study using a similar sorting strategy based on type II collagen gene expression has led to a much higher quality of iPSC-derived cartilage tissue [47]. One drawback of MACS is the requirement for a large starting cell number to obtain reasonable numbers of the enriched cell pool, for both gene expression analysis and 3D culture experiments. We posited that this CD24^+^ subpopulation could proliferate if reseeded onto a T75 flask and passaged thereafter to maintain a line of CD24^+^ iPSCs. However, that fraction did not readily expand in culture and was characterized by low viability in our pilot experiments (data not shown). Thus, the results currently support the concept of directed differentiation of sorted CD24^+^ iPSCs only immediately after selection. However, additional tests are needed to ensure that no ESC markers (i.e. Nanog) express in CD24^+^ cells in order to minimize any tumorigenic potential in the development of a therapeutic application. Furthermore, the differentiated CD24^-^ iPSCs also expressed some NP-associated markers suggesting that selecting iPSCs with MACS via CD24 only can enrich, but not uniquely select progenitors for NP cells. Nevertheless, the current study is a first step towards future work that may rely upon use of two or three markers for selecting iPSCs with potential to differentiate into NP cells of notochordal lineage.

Sakai and co-workers have suggested that Tie2 and GD2 expression are important cell-associated markers upstream of CD24 expression in NP progenitor cells, and that all of these markers may be used to sort cells for the NP progenitor populations in both human and mouse NP [14]. Tie2 is a tyrosine kinase receptor and is expressed in endothelial, haematopoietic and neural stem cells [68], while GD2 (disialoganglioside) is a component of the plasma membrane found mainly in the nervous system and is a marker for MSCs derived from both bone marrow and umbilical cord [69,70]. In the current study, we identified that about 10% of undifferentiated mouse iPSCs expressed Tie2 and 17% of iPSCs expressed CD24, but no expression of GD2 was detected in mouse iPSCs at the undifferentiated stage. It is possible that GD2 may be downstream of Tie2 and CD24 expression during mouse iPSC differentiation. Further studies that use sorting for Tie2^+^ or even GD2^+^ cells from iPSC populations at an early time point of differentiation for NP-genic progenitors may elucidate the functional roles and relationships of these three NP progenitor markers. Such an understanding could enhance methods to direct differentiation of iPSCs to the notchordal or NP cell lineage.

The mouse iPSCs used in the current study were generated via the use of viral vectors and characterized previously for iPSC-derived cardiac progenitor differentiation to cardiomyocytes [45]. In that study, the pluripotent potential of the undifferentiated iPSCs was confirmed through detection of pluripotency marker expression and generation of smooth muscle, cartilage and endothelial cells *in vitro* [45]. The ability of these iPSCs to form teratomas has not yet been tested [45], although teratomas were successfully formed from another mouse iPSC line derived using a similar protocol [47]. In the current study, we are interested in the potential of mouse iPSCs to differentiate into NP-like cells with notochordal lineage that we define through a subset of known cell-associated markers. This may represent the first demonstration of differentiation into cells of a notochordal lineage, and definition of culture conditions that may promote this differentiation for iPSCs. Our ultimate goal is to be able to identify a reliable fraction of NP-genic progenitors from iPSCs with efficient cell sorting and to use these progenitors in cellular therapy for disc repair.

In summary, this study evaluated a novel mouse iPSC cell source and its potential use for differentiation into a well-defined NP cell phenotype. The results demonstrate that an enriched CD24^+^ fraction of iPSCs can be directed toward an NP-like phenotype as evidenced by the expression of NP-like cell clustering morphology and accumulating NP-like matrix in the absence of growth factor supplementation. The results also demonstrate that unsorted iPSCs can be differentiated to an NP-like cell phenotype when cultured with supplemental conditioned media obtained from native NP tissues, and under hypoxic conditions. These studies are the first to report the expression of NP cell-associated markers in an iPSC cell type under undifferentiated and sorted (CD24^+^) conditions, and after spontaneous differentiation and in vitro culture. The findings lay the groundwork for future studies that can further refine cell sorting and culture conditions and definitions of the NP cell phenotype to advance use of iPSCs for intervertebral disc tissue repair and regeneration.
